# How do pig veterinarians view technology-assisted data utilisation for pig health and welfare management? A qualitative study in Spain, the Netherlands, and Ireland

**DOI:** 10.1186/s40813-024-00389-3

**Published:** 2024-10-10

**Authors:** Xiao Zhou, Beatriz Garcia-Morante, Alison Burrell, Carla Correia-Gomes, Lucia Dieste-Pérez, Karlijn Eenink, Joaquim Segalés, Marina Sibila, Michael Siegrist, Tijs Tobias, Carles Vilalta, Angela Bearth

**Affiliations:** 1 Consumer Behaviour, Institute for Environmental Decisions, ETH Zürich, Universitätstrasse 22, 8092 Zürich, Switzerland; 2 IRTA, Programa de Sanitat Animal, Centre de Recerca en Sanitat Animal (CReSA), Universitat Autònoma de Barcelona (UAB), 08193 Bellaterra, Spain; 3 Unitat Mixta d’Investigació IRTA-UAB en Sanitat Animal, Centre de Recerca en Sanitat Animal (CReSA), Universitat Autònoma de Barcelona (UAB), 08193 Bellaterra, Spain; 4 WOAH Collaborating Centre for the Research and Control of Emerging and Re-Emerging Swine Diseases in Europe (IRTA-CReSA), 08193 Bellaterra, Spain; 5 Animal Health Ireland, 2–5 The Archways, Carrick on Shannon, Co. Leitrim, N41 WN27 Ireland; 6 Royal GD, Arnsbergstraat 7, 7418 EZ Deventer, The Netherlands; 7 Departament de Sanitat i Anatomia Animals, Facultat de Veterinària, Universitat Autònoma de Barcelona, 08193 Bellaterra, Spain

**Keywords:** Qualitative study, Focus group, Veterinarians, Pig health, Pig welfare, Data utilisation, Technology

## Abstract

**Background:**

Application of data-driven strategies may support veterinarians’ decision-making, benefitting pig disease prevention and control. However, little is known about veterinarians’ need for data utilisation to support their decision-making process. The current study used qualitative methods, specifically focus group discussions, to explore veterinarians’ views on data utilisation and their need for data tools in relation to pig health and welfare management in Spain, the Netherlands, and Ireland.

**Results:**

Generally, veterinarians pointed out the potential benefits of using technology for pig health and welfare management, but data is not yet structurally available to support their decision-making. Veterinarians pointed out the challenge of collecting, recording, and accessing data in a consistent and timely manner. Besides, the reliability, standardisation, and the context of data were identified as important factors affecting the efficiency and effectiveness of data utilisation by veterinarians. A user-friendly, adaptable, and integrated data tool was regarded as potentially helpful for veterinarians’ daily work and supporting their decision-making. Specifically, veterinarians, particularly independent veterinary practitioners, noted a need for easy access to pig information. Veterinarians such as those working for integrated companies, corporate veterinarians, and independent veterinary practitioners expressed their need for data tools that provide useful information to monitor pig health and welfare in real-time, to visualise the prevalence of endemic disease based on a shared report between farmers, veterinarians, and other professional parties, to support decision-making, and to receive early warnings for disease prevention and control.

**Conclusions:**

It is concluded that the management of pig health and welfare may benefit from data utilisation if the quality of data can be assured, the data tools can meet veterinarians’ needs for decision-making, and the collaboration of sharing data and using data between farmers, veterinarians, and other professional parties can be enhanced. Nevertheless, several notable technical and institutional barriers still exist, which need to be overcome.

**Supplementary Information:**

The online version contains supplementary material available at 10.1186/s40813-024-00389-3.

## Background

Pig production has become one of the most economically dominant farming sectors in the European Union (EU), with pork ranking as the most consumed meat among terrestrial animals [1, 2]. To meet consumers’ demand for pig products and increase productivity in a cost-efficient manner, pig production has mainly been industrialised by shifting into intensive production in the EU [1]. Meanwhile, intensive pig production is facing multifaceted challenges such as public criticism of the healthiness and safety of pork, pig welfare, and environmental pollution [3], industry stakeholders’ concerns about the treatment of diseases, and the regulated antibiotic use in the EU [4–6]. Particularly, infectious respiratory disease, such as the porcine respiratory disease complex (PRDC), and gastrointestinal disorders are posing challenges to the pig industry, resulting in reduced efficiency of pig production, financial losses, and poor animal welfare [7–9]. Given that causes of infectious respiratory and gastrointestinal disease are most often multifactorial in nature, which are associated with various factors, such as complex pathogen interactions, environmental conditions, farming management strategies, and production systems [10–12], seeking effective interventions for disease control and prevention is of primary importance.

To face the abovementioned challenges of pig health and welfare management, data-driven decision-making in pig farming has been increasingly investigated in prior research [13, 14]. A variety of data collected on the farm, in the slaughterhouses or through diagnostic laboratories have been analysed by researchers to understand the spread of infectious diseases and to monitor pig health status [15–17]. For instance, previous studies have demonstrated the advantages of applying sensors and machine learning algorithms to early detect pig infectious diseases e.g., porcine reproductive and respiratory syndrome (PRRS) and African swine fever (ASF) [18, 19]. Nowadays, to improve pig production management, various technologies have been developed for pig farming to facilitate data collection, management, and analysis [20–23], namely pig monitoring systems (e.g., sensor-based monitoring of individual pigs), data management systems and decision support systems [15]. For example, precision livestock farming can be integrated into pig farming to monitor and track animals, to provide predictive analytics, and to support farmers’ decision-making by inspecting data, such as animal behaviour, feed intake, and coughing [23–25]. Moreover, information source platforms, such as national pig disease surveillance systems and websites providing pig production and health information, have been developed for industry stakeholders and veterinary practitioners to monitor the occurrence of endemic diseases, and thereby to control disease outbreaks in a timely fashion [26, 27].

While these innovative technologies exhibit benefits for animal production and disease prevention, notification and control, particularly with the provision of farm data [26, 28], previous studies have identified gaps between the generation and the utilisation of data among farmers, primarily due to the lack of skills in data analysis and interpretation, more reliance on own knowledge and experience instead of the use of technology, and the inherent limitation of technology itself [29, 30]. Nevertheless, veterinarians, as one of the most important stakeholders and trusted information sources for pig disease control, have rarely been the focus of prior research to explore their perspectives of data utilisation and technology usage, especially regarding pig health and welfare management [31, 32]. Therefore, there is a lack of an overall understanding of how veterinarians utilise data together with the application of technologies and what data-based digital tools veterinarians need to support their decision-making and improve the management of pig health and welfare.

Given that veterinarians’ roles might vary in different countries and farming systems, the current study aimed to explore veterinarians’ views of data utilisation and their needs for technologies and data in relation to pig health and welfare management in Spain, the Netherlands, and Ireland. Spain, as the leading pig producer in the EU, is mainly composed of large-scale production with vertical integration that supply feeds, provide veterinary technical services, and produce pigs from birth to slaughter [1, 33, 34]. The Netherlands, as the second biggest piglet producer in the EU, has a main orientation on pig and pork export [35]. Although Ireland is a relatively small pig producer, it has one of the highest average pig herd sizes in Europe [36]. In both the Netherlands and Ireland, veterinarians are often part of independent pig practices, who provide consultancy services to family-owned or family-operated farms. Thus, the objectives of present study were to understand veterinarians’ *status quo* of data utilisation using technology and to explore their needs for data utilisation with the application of technologies in these European countries, which may contribute to the user-oriented improvement of data tools for pig health and welfare management and support pig veterinarians’ decision-making [37].

## Methods

### Research design

This study applied qualitative research methods, specifically a series of focus group discussions, in three European countries (Ireland, Spain and the Netherlands) to explore and comprehend veterinarians’ views of data utilisation and technologies application for pig health and welfare management. Typically, focus group discussions can facilitate the identification of a broad range of perspectives on specific research topics by engaging interactive discussions among a group of participants [38, 39]. For this, a semi-structured interview guideline was developed in iterative discussion rounds with all authors. Semi-structured interviews are often preferable to structured and unstructured interviews, as it enables researchers to explore interviewees’ in-depth opinions by focusing on a list of research questions. It also allows researchers the flexibility to amend questions when interesting, surprising, or new topics emerge during the interview [40, 41]. Specifically, this interview guideline was composed of four parts (see Additional file 1). The first part focused on veterinarians’ goals in pig health and welfare management. The second and third parts aimed at understanding veterinarians’ views of using technologies and data for pig health and welfare management. The last part targeted veterinarians’ insights into the management of respiratory and gastrointestinal diseases and other challenges of health management. All focus group sessions were held in the language of the country, i.e. Spanish, Dutch, and English.

### Participants

Two focus group discussions were conducted in Spain (*n*_1_ = 6 participants; *n*_*2*_ = 6 participants), the Netherlands (*n*_1_ = 3 participants; *n*_2_ = 4 participants), and Ireland (*n*_1_ = 5 participants; *n*_2_ = 3 participants), respectively. In Spain, five participants were veterinarians employed by integrator companies, four were corporate veterinarians who provided distinct consultancy services (genetics, technology use, and economics) at farm level or for a non-profit national organisation of pig farmers. Two Spanish participants were independent practitioners, who provided consultancy services on pig health for multiple farms and companies, and the remaining one was a sectorial representative of a producers’ association at the national level. In the Netherlands, most participants were independent veterinary practitioners assisting privately-owned farms (e.g., family-owned farms) and one veterinarian worked for a feed company. In Ireland, most participants were independent veterinary practitioners working for privately-owned farms; one participant worked for a research organisation.

### Study procedures

The focus group discussions took place between June and November 2022 in Spain, the Netherlands, and Ireland. Before the focus group discussions, all participants were asked to sign an online informed consent form to assure permission to record the focus group discussions and analyse data anonymously. Data were collected either through face-to-face discussions or through online meeting discussions, according to the flexibility of the schedules and availability of participants. To guide the focus group discussions, moderators used a semi-structured interview schedule to ask a series of open-ended questions. The moderators in each country were native speakers or fluent in the respective language, who have a rich experience in pig health and welfare management. Each focus group discussion lasted around 90 min. All focus group discussions were recorded, transcribed, and translated into English with the assistance of the moderators and a professional transcription company. This study was approved by the Ethics Commission of the Swiss Federal Institute of Technology Zurich (approval number: EK 2021-N-224).

### Qualitative analysis

The transcribed data were analysed using thematic analysis [42, 43]. Initially, the first author repeatedly read the transcripts to get familiar with the data. Then, an inductive approach was applied to derive a series of codes (e.g., a word or short phrase) from the data, and the codes were refined based on the research questions in a deductive manner. Specifically, codes regarding the two main research questions, the *status quo* of veterinarians’ data utilisation using technology, and veterinarians’ needs for data and data tools to optimise data utilisation for pig health and welfare management were developed. Further, codes were categorised to form themes and subthemes which represented patterns of sharing meaning across the data set. Finally, the study results were discussed and revised to ensure each theme and subtheme were clearly defined and explained by the authors (some of authors were moderators of focus groups in the current study). The qualitative analysis was performed with the software Nvivo (Release 1.6.2, QSR, International). The transcripts of the participants’ discussions were anonymised before analysis. All themes and subthemes are shown in the section of results in a hierarchical list where main themes were ordered alphabetically, followed by the subcategory for subthemes. Quotations are presented in italics and participants were anonymously numbered according to the sequence they presented in the transcripts together with the information of focus group number in each country and the country of origin. For example, *Spanish FG 1, RES 1* indicates that this participant was respondent 1, who participated in the focus group 1 in Spain.

## Results

The analysis of the focus group discussions resulted in six main themes and thirteen subthemes, which are shown in Table 1. These themes and subthemes address the two research topics introduced previously: (1) the *status quo* of veterinarians’ data utilisation using technology, and (2) veterinarians’ needs for data utilisation together with the application of technologies.

**Table 1:** Summary of main themes and sub-themes according to research topics

Research topic	Theme	Sub-theme
The *status quo* of data utilisation using technology	a. Varying levels of technology use	
	b. Role of technology in pig health and welfare management	
	c. Gap between data and data utilisation	
Veterinarians’ needs for data utilisation together with the application of technologies	d. Data requirements	Longitudinal data
		Timely data
		Reliable data
		Standardised data
		Contextual data
	e. User experience	Easy to use
		Easy access to pig information
		User adaptability
		Data centralisation
	f. Information output	Monitoring pig health and welfare
		Information sharing
		Intuitive decision support
		Early warning

### The *status quo* of data utilisation using technology

- Varying levels of technology use

In the current study, the status of applying technology for data utilisation varied among participants across countries and within countries. In general, participants use programmes, such as MS Excel™ or Power BI, or other types of online data platforms and mobile device applications to access, process, analyse, and visualise data that has been collected from farms, slaughterhouses, and laboratories. Specifically, participants often use these data tools to understand data patterns and identify potential health problems of pigs. One participant talked about the use of the data management tools: *‘We use management programs to analyse production parameters and, depending on the results, or if we can observe any deviation of parameters, we can focus on whether we really have a health problem or not’. (Spanish FG 2, RES 10)*. Some participants in Spain and the Netherlands use software applications as alarm tools to receive notifications from the farm when unexpected changes of data, such as abnormal environmental parameters, feed and water consumption, weight, and mortality, are detected. For example, to manage respiratory disease, one participant used the alarm tool by *‘telling the system to let me know when mortality exceeds 4% in piglets, or when it exceeds 3% in deaths due to respiratory issues.’* *(Spanish FG 1, RES 6)*. Additionally, the advantages of using mobile communication platforms for telemedicine were highlighted by some participants from Ireland and Spain. Particularly, mobile communication platforms allowed veterinarians to quickly check the pig health status by receiving information, such as pictures or videos from farmers without the need or before an in-person visit.

Despite various data tools mentioned by pig veterinarians, tools such as data management software and alarm tools are not widely and equally employed by participants for pig health and welfare management across and within different countries. A technology divide in Spain was found between independent veterinarians who provided services to privately owned farms: *‘We basically only work with production parameters in fattening, but technological management tools on farms, we don't have them.’ (Spanish FG 2, RES 8)* and those who were employed by pig companies: *‘When I visit a farm, I use the tablet and I get information on the farm of origin of the pigs, number of the batches, feed consumption and so on…we have it all. All of that is in the database.’ (Spanish FG 1, RES 3)*. Some independent participants still use their own spreadsheet program to record, analyse, and visualise data, which was not specifically designed for pig health and welfare management. However, others, especially veterinarians that are employees of an integrated pig company, have already been using company-owned mobile device applications and management software to access farm data, to visualise disease by the farm of origin, and to receive alarm notifications when farm abnormal data were detected.

b. Role of technology in pig health and welfare management

Although technological tools that are specially designed for pig health and welfare management have not been widely available to participants in the current study, the participants generally held a positive attitude towards these technologies and believed that using data tools was a beneficial approach to manage pig health and welfare, especially for the management of infectious respiratory and gastrointestinal diseases. In summary, data tools are recognised for their effectiveness, efficacy, and economic benefits in evaluating pig health and welfare status, improving production, and supporting veterinarians’ decision-making.

However, some participants pointed out that applying technology could not replace farmers’ observation, governance of farm, farmers’ compliance and feedback on veterinarians’ advice, and veterinarians’ clinical experience in pig health and welfare management. One participant explained: *‘The stockman is the first person to see the ill health in their animals, so I suppose that's one of their most basic jobs, just basically using their eyes and ears and looking at the pigs…. No matter how advanced your technology is, that will always be important.’* *(Irish FG 1, RES 23)*.

c. Gap between data and data utilisation

Participants believed that a good utilisation of data could provide them with useful information to monitor and evaluate pig health and welfare status, benchmark against the farm’s past or other farms’ performance, detect potential disease risks, and develop a plan of action for therapeutic or preventative procedures. Nevertheless, data seemed not to be effectively and efficiently utilised to assist participants’ decision-making in pig health and welfare management: *‘We almost hear it with the same words in different places and very, very different countries. Not only small or medium-sized companies, but also large or very large companies. You hear: “we are rich in data, but poor in information”’.’* *(Spanish FG 1, RES 6)*. Several limitations of the current data tools were highlighted by veterinarians. For instance, many participants stated that they had to collect data from data sources or request data from farmers, and then migrate the data into their own software for data analysis and visualisation, which was perceived as time-consuming and laborious. Notably, some Spanish participants expressed their apprehension about the limitation of software programs, such as MS Excel^TM^, given these tools are not specifically designed for pig health and welfare management. Additionally, participants mentioned that the current function of data tools were not able to continuously track pig health-related data from the farms, slaughterhouses, and the laboratories, which failed to provide them a complete picture of the pig health status. For one thing, there is a lack of an integrated data tool that links different data sources. For another, continuous data collection from multiple sections, such as farms, slaughterhouses, and laboratories, has not yet been achieved, at least within the investigated regions. Although some digital tools such as sensors have been employed on farms to automatically generate data, it still requires farmers to manually collect data and transfer data into the management for data storage and processing, which is beyond farmers’ working routines. Furthermore, participants mentioned that the current data tools, such as alarm systems, were lacking decision-support functions to treat potential risks of pig health, based on abnormal data, which impedes timely disease control.

### Veterinarians’ needs for data utilisation together with the application of technologies

**Fig. 1:**
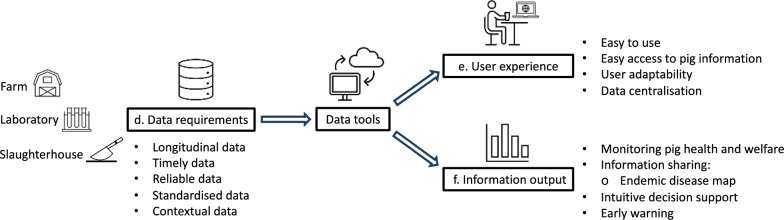
An overview of factors that may facilitate data utilisation based on participants’ need for data and data tools

To fill the gap between the raw data and data utilisation, participants expressed their needs for both data and data tools that might facilitate them to use data for pig health and welfare management. An overview of pivotal factors that contribute to data utilisation can be found in Fig. 1. Specifically, these factors represent themes and sub-themes that demonstrate how data tools fulfil participants’ needs for data utilisation in terms of data requirement, user experience, and information output, which can be tracked back in Table 1.

d. Data requirements

Participants valued the role of data in pig health and welfare management because of its potential benefits in understanding pig health status, establishing diagnoses, and taking interventions to control diseases. However, the quality and availability of data impacted participants’ data utilisation, prompting them to discuss their requirements for data.

#### Longitudinal data

Participants highlighted the importance of using longitudinal data for pig health and welfare management. As an example of using longitudinal data, one participant said: *‘Laboratory results would be historical data for the farm, would be something that gives you a background or benchmark to use for the current visit or future visit.’ (Irish FG 1, RES 23)*. Specifically, participants believed that they could get a comprehensive overview of the situation in the pig herds by inspecting the continuously recorded data over time from pig farms, laboratories, and slaughterhouses. For instance, participants could analyse longitudinal data to characterise data patterns and identify potential health risks by comparing data at different time points or on different farms within the same period. To utilise longitudinal data, tracking the progress of treatment for pig diseases (e.g., therapy compliance and use of medicine) was also highlighted as a good strategy by some participants.

However, participants pointed out that, in many cases, data that comes from pig farms (e.g., disease treatment, mortality, behaviour, cough, and environmental parameters), slaughterhouses (e.g., records about ante-mortem and post-mortem inspection), and laboratories (e.g., results of sample tests) are not being recorded and made available continuously thus far.

#### Timely data

The timeliness of updating pig health-related data were stressed as another essential data issue because it enabled participants to obtain a quick evaluation of pig health status and make timely decisions to control diseases accordingly. One participant stressed: *‘When we talk about diseases, the first thing is punctuality, knowing what is happening on a farm, because after a week it may already be too late.’ (Spanish FG 1, RES 1)*. However, current data tools seem not to fulfil participants’ needs for timely data, since many farmers still manually upload data to the data system, postponing data updates and data sharing with veterinarians. Moreover, due to the lack of internet connectivity on some farms, farmers cannot update and share data with veterinarians in a timely manner.

#### Reliable data

Getting reliable data was stressed as one important step for data utilisation by participants. For instance, the objective information extracted from reliable data on pig farms could support veterinarians’ decision-making and benefit their collaborations with farmers. Some Irish participants explained that the current data tools might not guarantee the accuracy of the collected data because of potential errors caused by manual data input. If data could be automatically recorded and updated to the data tool, veterinarians could then get more objective and more reliable data to assess the pig health status and make decisions, as suggested by one participant: *‘If you had these kind of things recorded automatically without you even going to the farm and not taking any effort, then you go back to them (farmers) and say： “Look, it's actually happening or not.”’ (Irish FG 2, RES 26)*.

#### Standardised data

The lack of standardised data may lead to variability in data recorded. For instance, one participant expressed his worries of using slaughterhouse data: *‘Because you're using four or five vets (in the slaughterhouse), the way of scoring the lesions would be completely different. It's not saying that one vet is better than another one, but it's open to misinterpretation.’ (Irish FG 1, RES 25)*. If data could be measured and collected in a consistent manner, e.g., using the same scoring system and the same data tools, veterinarians could obtain objective, robust and tracible data to compare health-related data and make informed decisions. Indeed, some Dutch participants stressed the value of using standardised data to follow up disease treatments and plan actions of health management. To standardise data and enable data sharing for pig health management between farmers and veterinarians, some Spanish participants suggested applying the same digital tools to generate, collect, record, and calculate data, which might be an ideal solution in the future.

#### Contextual data

Consistently, the loss of context could lower the perceived usefulness of data and increase the misinterpretation of the data among participants. Some Spanish participants explained the necessity of having background information when analysing data. For example, mortality, measured at different time slots during the pig production, could be interpreted differently, which significantly affects the next step of health management for pigs. To make a more accurate assessment and to get a clearer understanding of the pig health status, some Dutch participants suggested getting more detailed data recorded during the inspection of the intestinal tract in the slaughterhouse. For instance, one participant stated: *‘There is polluted data (e.g., noisy data, biased data, not reliable data) in the slaughterhouse. They weight the intestinal packages, they weight the content to check whether it is full or not full, but actually there might be more data to get out of it about how long it is or what the diameter of that is or how big that stomach is or something, that also says something about health.’ (Dutch FG 2, RES 17)*.

e. User experience

To increase the efficiency and effectiveness of utilising data, several important features of data tools were discussed according to veterinarians’ experience of using data tools. Specifically, data tools should be easy to use, easy to get pig information, and adaptable to users’ preference by integrating different types of data in one platform. Although these insights might seem trivial, it is worthwhile to list them here, as these aspects are not always considered in the development of data tools according to the participants.

#### Easy to use

Although some data tools have been applied to visualise and analyse data, participants stated that these tools were too complicated to use, which failed to encourage them to continuously use the data tools. One participant explained: *‘The important thing with a lot of those tools is that it should be labour-friendly…Of course, we have often launched all kinds of new things, but they sometimes die a silent death because it is all too labour-intensive, too complicated. You have to keep guarding against that every time.’ (Dutch FG 1, RES 15)*. Therefore, data tools should ensure the ease of operation and the information conveyed by data tools should be easy to understand, as suggested by participants.

#### Easy access to pig information

To utilise data, participants, especially those who were part of independent practices, mentioned that they often requested data via multiple ways, examples including in-person farm visit, email, or telephone message. These inconvenient, unsystematic, and oftentimes unsecured ways of obtaining data disabled veterinarians to have a timely and comprehensive vision of pig health status, and in turn, influenced veterinarians’ decision-making and action-taking of disease control and pig health and welfare management. Thus, veterinarians desired to have a data tool that provides easy access not only to raw data but also to useful information that can be visualised or easily understood.

#### User adaptability

A data tool that is adaptable to users’ habits and working routines may increase the efficiency and effectiveness of data utilisation, as noted by participants. For instance, rather than spending a lot of time inspecting all data from farms, only allowing health related data to be directed to veterinarians could enhance their data utilisation. An example can be found from the perspectives of this participant: *‘Let's think about how to make an information management system that is valid for the person who is on the farm, that is valid for the veterinarian, that is valid for the management, because they have different needs, but the same effort should apply to everyone.’ (Spanish FG 1, RES 6)*. Moreover, the flexibility of using a data tool for data analytics was emphasised by participants. Some veterinarians preferred to have multi-functions (e.g., monitoring, data analysis, visualisation, early warning) into one platform, while some preferred to analyse data by themselves using their preferred data tools.

#### Data centralisation

Some participants from Spain and the Netherlands indicated that they lacked a data tool that integrated different types of data for a quick overview of the data in relation to pig health and welfare: *‘Things (data) come in from all sorts of sides, but if you have a central dashboard—for a farmer as well—that we can look at, then we'll really be able to take steps, where you link it all together, but that's what's missing’ (Dutch FG 1, RES 14)*. If all data could be centralised into one platform, e.g., linking farm data with slaughterhouse data and laboratory data, participants could make a comprehensive assessment of pig health and welfare status, which could contribute to the planning of the schedule of farm visiting, and the plan for treatment.

f. Information output

To assist the day-to-day task and timely control of pig disease, participants suggested that the data tools should provide insightful information that helps users to understand the status of pigs, early detect specific diseases, and provide data-driven decision-support.

#### Monitoring pig health and welfare

A data tool that tracks the status of pig health and welfare over time in parallel to management changes or potential health hazard events was perceived by participants as time- and labour-saving, and beneficial for the management of diseases, especially regarding the management of infectious respiratory and gastrointestinal diseases. One participant explained: *‘To establish cause and effect of acute health problems, you can link them with specific events in the last week or two weeks, but four months later you have already forgotten about these events. If you could do a little more (data recording) over time, it would be easier to take a long-term approach for health management.’ (Dutch FG 2, RES 16)*. Specifically, some participants preferred to visualise long-term trends about pig health and welfare (e.g., mortality, disease treatment, environmental control, feed and water consumption, behaviour) from farms, laboratory, and slaughterhouse, ideally updated daily or in real time. In this way, veterinarians can assess the status of pig health and welfare and track the process of disease treatment in both the short term (e.g., at real-time level) and long term, and then they can adjust their action plans quickly and accordingly.

#### Information sharing

Participants believed that the more health-related information could be shared from pig farms, the more benefits would emerge for pig health and welfare management, especially in controlling infectious respiratory and gastrointestinal diseases. Some Spanish participants wished to have a centralised access to an ‘endemic disease map’ to control the spread of infectious diseases by encouraging the sharing of data, such as origin of animals, entry route, disease incidence and mortality by region, and mortality cause between farms, veterinarians, and other professional parties. For instance, a pig health monitoring system developed by a non-profit organisation for providing an epidemiological map of diagnoses in north-eastern Spain was highly valued by Spanish participants, as it allowed veterinarians to examine the disease prevalence by region in an anonymous way (information about this web application, GSP monitoring system, can be found via the link http://www.gsplleida.net/es/content/app-del-gsp). However, such a monitoring system has not yet been widely applied into specific actions related to pig health management.

Several potential barriers to data sharing were discussed by participants. The low awareness of the importance of sharing data and the intense competition between farms and companies might account for industry stakeholders’ low willingness of sharing data with external stakeholders, as explained by some Irish and Spanish participants. Implementation of regulation and training of reporting data might drive pig industry stakeholders to share more pig health-related data, as suggested by some Spanish participants. Moreover, some Dutch participants pointed out that lacking a standardised way of inputting data may impair the usefulness of the disease monitoring system at a national level via sharing data for pig health management. Additionally, the low trust in the quality of shared data was a potential barrier to share data with external stakeholders, as explained by some Spanish participants. If data can be collected and recorded in the same manner e.g., use the same digital tool and the same measurement method, the credibility of shared data may increase. Furthermore, some Irish participants explained that privacy concerns about sensitive data (e.g., finances and medicines) could be another barrier to sharing data from pig industries with external parties especially the data about notifiable diseases. One participant stressed*: ‘That's sharing data. You can't share, it's just personal. Unless you give to a farmer an individual number and you do a benchmarking, and you are the only one and the farmer knows his number.’ (Irish FG 2, RES 25)*. If data can be shared and used anonymously e.g., benchmark against other farms, farmers might be more willing to share data.

#### Intuitive decision support

Given that rich data are now available to be collected from farms with the use of technologies, participants discussed their further needs for advanced data tools regarding the utilisation of big data. Data tools that perform intuitive decision support to improve pig health and welfare management were perceived as useful by participants. For instance, one participant suggested: *‘The tools will improve a lot by integrating data, by generating artificial intelligence to be able to build strategies for health improvement in pig flows.’ (Spanish FG 2, RES 7)*. This idea was found among Dutch and Spanish participants, and they believed that artificial intelligence (AI) could be applied to conduct exploratory and explanatory analytics, aiming to provide suggestions for users to facilitate their decision-making and action-taking.

#### Early warning

Participants agreed that the employment of early warning tools could provide urgent decision-making support and help users to take timely action for disease control and prevention, especially concerning the management of infectious respiratory and gastrointestinal diseases. Specifically, data such as mortality, fecal score, feed intake, water consumption, use of medicine, weight, cough counts, and environmental parameters of the pigs could be treated as health indicators to generate alerts, as suggested by participants. While some alarm tools have been employed on farms, participants from Spain and the Netherlands underlined that these alarm tools lacked the function of predicting health risks and failed to aid with action-taking. Therefore, current alarm tools have not met veterinarians’ needs yet. Furthermore, some Spanish participants highlighted that the early warning should be flexible to use, allowing users to set individual thresholds triggering a warning.

Furthermore, participants discussed their preferences for the alarm distribution (i.e., which alerting information should reach whom) when they used early warning tools. Some participants suggested that the responsibility of each stakeholder should determine who receives the alarms. One participant suggested that: *‘You manage it (water consumption) separately or you can have an API (application programming interface) to connect them in a way that you have all data together and receive the alerts. These are received either by the farmer or by the veterinarian, or by the manager, depending on his/her responsibility.’ (Spanish FG 1, RES 6)*. Specifically, farmers were suggested to receive alarms when abnormal changes of production data, such as feed or environmental data, were detected, and veterinarians were encouraged to receive alerts when emergency pig health issues such as high mortality were identified. Nevertheless, conflicts about who should receive an alarm emerged among some Irish and Spanish participants. For instance, some Irish participants who were independent practitioners working for privately-owned farms explained that veterinarians’ routine job was to diagnose diseases and guide treatments, which was usually initiated by farmers, who have the best understanding the health status of their pigs. Thus, alarms should be checked first by farmers and then, be forwarded to veterinarians. Inversely, some Spanish participants preferred to receive the alarms directly, as they needed to get a timely and objective evaluation of the pig health status to control and manage the pig disease.

## Discussion

This study uncovered the gap between data and data utilisation for pig health and welfare management from the perspectives of pig veterinarians in Spain, the Netherlands and Ireland. A discrepancy of technology application for data utilisation was identified between veterinarians who are independent practitioners and those who were employees of pig companies. Overall, veterinarians’ needs for data utilisation have not been fully met with the application of the current technologies.

When it comes to the data utilisation in livestock farming, the influencing factors are often related to individual characteristics e.g., personal data analysis skill and the user-friendliness of the available data tools [30, 44, 45]. However, the foundation of data utilisation for pig health management, namely the availability and quality of the data itself, might be overlooked. The current study revealed veterinarians’ different needs for data. Specifically, pig farms and companies, laboratories, and slaughterhouses were important data sources, but in reality, frequently fall short of providing timely data to facilitate veterinarians’ decision-making. The current findings kept consistent with previous studies that timely feedback on disease syndromes and quick information update about the disease trend contributed to the pig health surveillance and management, from the perspectives of Canadian veterinarians [46]. Similar results can be observed in the study of Alarcon et al. [26], as British pig farmers perceived some online platforms providing pig disease information as useless if these platforms failed to update information in a timely manner.

Data standardisation and data reliability were other concerns underlying the discussion on data utilisation raised by pig veterinarians. Veterinarians noted the importance of standardised data for pig health assessment, disease treatment, and action planning by exemplifying the adverse outcomes caused by unstandardised ways of measuring data in the slaughterhouse and reporting data on the pig health surveillance system. In line with the current study, previous studies have revealed the lack of data standardisation for meat inspections at the EU level [47–49]. Without a standardised way of measuring and recording data during the meat inspection in slaughterhouses, the quality of data may drop and in turn affect the accuracy of pig health assessment [48, 50, 51]. It is noteworthy that a lot of data is still required to be manually transferred to the data management tools, which could impact the reliability of the data and decrease the efficiency of using data, as mentioned by veterinarians in the current study. Additionally, the lack of contextual information was found to be an important factor affecting data interpretation and assessment of pig health by veterinarians. Indeed, Sarikaya et al. [52] pointed out that contextual information was needed to help users to understand the meaning of the visualised data (e.g., data trend visualised by bar chart) generated by the dashboard. To streamline the data utilisation for long-term pig health monitoring, application of information and communication technologies, such as smart sensors into precision livestock farming system, may improve data quality, speed of data collection, and the standardisation of data sets [53, 54]. To date, some sensors are commercially available to effectively and efficiently obtain data on the farm such as feed and water intake, environmental data, and pig coughing [53, 54]. However, the implementation of such technologies may face challenges of linking it with the existing data management software [53], the lack of internet connection on farms, high investment costs, and need for staff training associated with the technology application [55]. Still, additional efforts are required to standardise the way of recording data by including more details related to pig health, such as providing personalised training and guidance in the used terminology and format of the recorded data [48, 50].

Although rich data is generated and collected on pig farms, in slaughterhouses, and in laboratories, veterinarians in the current study stated that data had not been translated well into useful information. Should this change, this information could potentially support their decision-making in pig health and welfare management. To utilise big data for pig health and welfare decision support, data should be filtered or condensed to smaller subset of information that meet decision-makers’ information processing capacity [15, 56, 57].

Nowadays, many dashboards (not specific for pig health management) are available for benchmarking and to facilitate users to identify areas of focus in various fields, which can be categorised into operational ones (i.e., used at a real-time level) and strategic ones (used over a period) [52]. Consistently, veterinarians in the current study wished to monitor and visualise pig health data over time and ideally, in real-time to get useful information for benchmarking and early detection of potential risks for disease control. For this, quantitative data on for example mortality, food and water intake could be visualised in time series. These findings aligned with the study by Koltes et al. [58] that the visualisation function of a data tool may enhance user experience and promote technology adoption. However, it should be noted that as a pre-condition, multiple challenges related to the collection and recording of the data as discussed above need to be overcome. Veterinarians also expressed their need for monitoring pigs regarding qualitative data such as behaviours to facilitate health management. Although some of them mentioned that technology cannot replace individuals’ observations for pigs [59], continuous human observation in large-scale pig production is not feasible. The application of sensor-based cameras to monitor the changes of pig behaviours may enable the early detection of potential problems such as tail biting to improve pig welfare and facilitate farm staff to plan actions [60]. Overall, this was seen as a good solution for remote decision-making without frequently visiting a farm in person [54].

The veterinarians’ views on data utilisation and their need for data tools was related to their specific roles in the three countries of investigation. Compared to veterinarians employed at integrator companies in Spain, the independent veterinarians lacked convenient access to pig health-related data and access to company-owned data management software. In most cases, independent veterinarians used their own programs to visualise and analyse the data they received through various means. Frequently, they may lack training in more complex data management tools, specifically designed for health-related data. Indeed, a digital divide has been observed as a factor affecting technology application in livestock farming between users who lacked technical skills and trained technical users [24]. Offering training in the use of data tools, allowing for time investments, and prior skills in data analysis may contribute to veterinarians’ data utilisation. Differences were also observed in terms of access to the data and desired output. For instance, independent veterinarians in Ireland preferred an early-detection alarm system to be filtered first by farmers because it was beyond their work routine to spend time examining the data and the alarm. The study of del Rocio Amezcua et al. [46] supported the current finding that it was laborious for practising veterinarians in Canada to consistently record data in a pig surveillance system because of the heavy workload. Current workloads and linked information processing capacity may constrain veterinarians from extracting useful information from data through individual data analysis. This might be especially true for the independent veterinarians that provide services to multiple farms. Instead of displaying extensive raw data to veterinarians, a data tool should provide timely and intuitively visualised information to help veterinarians easily understand the animal status, saving their labour and time costs. Furthermore, AI-based tools that reduce the cognitive load and support decision-making through providing suggestions might therefore be useful, according to the perspectives of veterinarians. So far, a variety of studies have investigated the application of machine learning algorithms for decision-making in pig farming, especially related to disease, feeding strategies and DNA analysis [54, 61]. However, whether AI tools can produce useful information to assist pig veterinarians’ action-taking remains to be explored in the future [61]. Lastly, Spanish veterinarians valued the geographical surveillance map to visualise the prevalence of infectious respiratory and gastrointestinal disease, which can be jointly used by pig health professionals. This was also found in prior studies that stressed the importance of a monitoring system for infectious disease control in Spain [62]. In light of the above, customised dashboards that are adaptable to different users’ needs for data utilisation should be one the of main considerations, which was also highlighted by prior research about the development of dashboards [52, 63, 64].

Not surprisingly, veterinarians in the current study wished to have a user-friendly tool to ease their data access, processing, and analysis. Many previous studies have uncovered the importance of user friendliness for technology application in livestock farming [30, 44, 65]. However, it seems that this principle is not consistently translated into practice yet. For instance, the study of Hartung et al. [30] illustrated farmers’ concerns about using a complex precision livestock farming system. To ease the data utilisation, farmers preferred all data to be displayed and managed in an integrated way. The current study kept consistent with prior studies that veterinarians needed a user-friendly tool that can link all the relevant data about pig health and welfare from multiple data sources, which was perceived as time- and labour-saving. Applying protocol-based application programming interface (API) has shown its benefit by connecting Internet of Things (IoT) devices (e.g., sensors) and data cloud platforms, which may contribute to the design of a user-friendly and integrated data tools that supports decision-making in livestock farming [66, 67].

To improve data utilisation for pig health and welfare management, increased collaborations are needed to strengthen the trust and data sharing between farmers, veterinarians, and other professional parties. Many prior studies have highlighted the benefits of building up a surveillance system for disease control and prevention, but it required the support from multiple stakeholders such as governments, laboratories, veterinarians, and industry [62, 68, 69]. Consistently, veterinarians in the current study stressed the importance of sharing data to prevent and control endemic infectious diseases. However, low awareness of the need to share and concerns over competition and data privacy might hinder the pig industry from sharing pig health-related data with external stakeholders [70, 71]. Rodríguez-Prieto [72] found that very little information about endemic diseases was shared on a surveillance system in Spain, thus, it contributed little to the decision-making in pig health management at population level. To visualise the distribution of endemic diseases at population level, support decision-making and enhance collaboration within the pig sector in Spain, Alba-Casals et al. [27] investigated a web application. This application was co-created by multiple experts such as computer scientist, researchers in pig disease and veterinarians, which allowed industry stakeholders and veterinarians to monitor endemic diseases in near real-time by requiring veterinarians to report data on it. To encourage the data reporting, Alba-Casals et al. [27] recommended trainings and regular exchanges on the benefits of sharing data on surveillance systems. Similar strategies were suggested by the veterinarians in our study to increase awareness and willingness to share.

This study has two limitations. Firstly, the focus group discussions were moderated by researchers specialised in the field of pig health but varying in disciplines, countries, and languages, which might cause bias when analysing the transcribed and/or translated data. However, the quality of transcribed and/or translated was revised by the moderators, and the themes and subthemes were discussed among the authors to reduce this bias. Secondly, the utilisation of data for pig health and welfare management requires the collaboration between different stakeholders e.g., pig industry, veterinarians, laboratories, slaughterhouses, and the government, but this study only included the veterinarian’s perspectives. Future studies should include different types of stakeholders to uncover their need for data utilisation with the application of technologies. Ideally, data tool developers keep the pre-conditions and needs of these different stakeholders in mind and engage in a regular exchange with the end users of their products. While this study provided some general insights into the needs for data tools, specific tools should be user tested to ensure their successful implementation.

## Conclusion

Results of this study reveal that although rich data related to pig farming is being collected and recorded from pig farms and companies, slaughterhouses, and laboratories, data is currently not yet utilised efficiently and effectively with the application of data tools for pig health and welfare management by veterinarians. A discrepancy of data access and technology use were found between some independent veterinary practitioners and company employed veterinarians. To support veterinarians’ decision-making, tool developers should not only consider the usefulness and usability of the data tool itself but also ensure the quality and accessibility of data requiring joint efforts from all sides. It is also essential to address users’ concerns about data sharing and meet their needs depending on their roles in pig farming. This might be achieved by conducting context-specific investigations to develop data tools that are tailored to meet veterinarians’ diverse needs for data utilisation. In the future, customised data tools with the application of advanced information and communication technology might be a solution to support veterinarians’ decision-making and their daily management of pig health and welfare.

## Supplementary Information

Supplementary Material.

## Data Availability

The data generated and/or analysed during the current study are not publicly available due to reasons of confidentiality.
