# Supplementary material for: How do pig veterinarians view technology-assisted data utilisation for pig health and welfare management? A qualitative study in Spain, the Netherlands, and Ireland
Source: Porcine Health Manag. 2024 Oct 10;10:40. doi: 10.1186/s40813-024-00389-3 (PMC11468428; doi:10.1186/s40813-024-00389-3)
Supplement: Supplementary file 1 — Supplementary Material. [file 40813_2024_389_MOESM1_ESM.pdf]

Additional file 1-Supplementary Material

How do pig veterinarians view technology-assisted data utilisation for pig health and welfare management? A qualitative study in Spain, the Netherlands, and Ireland

Xiao Zhou[1*], Beatriz Garcia-Morante[2,3,4], Alison Burrell[5], Carla Correia-Gomes[5], Lucia Dieste-Pérez [6], Karlijn Eenink[6], Joaquim Segalés[3,4,7], Marina Sibila[2,3,4], Michael Siegrist[1], Tijs Tobias[6], Carles Vilalta[2,3,4] & Angela Bearth[1]

[1]Consumer Behaviour, Institute for Environmental Decisions, ETH Zürich, Universitätstrasse 22, Zürich, 8092, Switzerland

[2]IRTA. Programa de Sanitat Animal. Centre de Recerca en Sanitat Animal (CReSA), Universitat Autònoma de Barcelona (UAB), Bellaterra, 08193, Spain.

[3]Unitat Mixta d'Investigació IRTA-UAB en Sanitat Animal, Centre de Recerca en Sanitat Animal (CReSA), Universitat Autònoma de Barcelona (UAB), Bellaterra, 08193, Spain.

[4]WOAH Collaborating Centre for the Research and Control of Emerging and Re-Emerging Swine Diseases in Europe (IRTA-CReSA), Bellaterra, 08193, Spain.

[5]Animal Health Ireland, 2–5 The Archways, Carrick on Shannon, Co. Leitrim, Ireland N41 WN27.

[6]Royal GD, Arnsbergstraat 7, 7418 EZ, Deventer, the Netherlands.

[7]Departament de Sanitat i Anatomia Animals, Facultat de Veterinària, Universitat Autònoma de Barcelona, Bellaterra, 08193, Spain.

*Corresponding author: Xiao Zhou, Consumer Behaviour, Institute for Environmental Decisions, ETH Zürich, Universitätstrasse 22, 8092 Zürich, Switzerland. E-mail address: xiao.zhou@hest.ethz.ch

Focus group discussion guide (English version)

Welcome and introduction (10 mins)

Moderator and assistant introduce themselves and the research purpose. Then each participant was welcome to briefly introduce themselves.

Focus group questions

Goals as a pig veterinarian (20 mins)

- **Thinking about pig health and welfare, what are your goals as a pig veterinarian for long-term and short-term?**
- **Why do you think this goal is important to you?**
- **How do you think technology and data could help you to achieve your goals?**

Technology use (20 mins)

- **What are your experiences of using tools and technologies related to pig health and welfare management?**

  - What technologies help with identifying or controlling diseases in pigs (if any)*?
  - How do the tools affect the decisions you make?
  - How do tools/technology impact your veterinary business?
  - What technologies would you like to see more widely implemented on farms to aid with pig health and welfare management?

- **In your experience as a pig veterinarian, what are the most useful aspects of the technologies you use?**

  - Why is [value/aspect] important to you?
  - How could the current technology be improved?

Data use (20 mins)

- **What is your experience of collecting and using data from pig farms?**
  - What types of data do you collect on the farms?
  - How do you collect this data?
  - What data do you collect without the use of technology? Can you explain why you use this way to collect data without the application of technology?
  - How do you utilise the data you collect?*
  - Who do you share the data with? And why?
  - How do they utilise your data?
- **How does the availability of data on a farm impact on the way you make decisions, if at all?**
  - Which types of data are most useful for you?
  - Why do you think this data is useful?

Respiratory diseases and gastrointestinal disease management (15 mins)

In the next section, we would like you to focus on the management of respiratory disease and gastrointestinal diseases.

- **What is your role in disease management on pig farms?**

- What helps you to diagnose the disease and come up with a treatment plan?
    - What types of data help you to diagnose the health problem?
    - Can you describe any tools/technologies that help you? If not, how do you think technology could help you diagnose the health problem?
- What role do your clients have in disease identification?
- How do you make joint decisions with your clients?
- What is the most important advice that you give your clients around preventing and managing respiratory and gastrointestinal disease on their farm?

- **How has your advice around respiratory and gastrointestinal disease management changed over time, if at all?**
    - What made you change your advice? *
    - Why do you think your current advice is better?
    - If there have not been any changes: why do you think your practices have stayed the same?
- **What other diseases do you consider a big problem on pig farms?**
    - Why is this disease a problem?
    - What helps you to diagnose the disease and come up with a treatment plan?

Wrap up (5 mins)

- **What are you taking away from today's discussion?**
- **Are there any final thoughts that anyone would like to share, before we close today's session?**

Moderator and assistant thank for every participant's contribution and close the discussion.
